# Long‐term follow‐up seizure outcomes after corpus callosotomy: A systematic review with meta‐analysis

**DOI:** 10.1002/brb3.2964

**Published:** 2023-03-16

**Authors:** Xiaolong Wu, Siqi Ou, Huaqiang Zhang, Yuhang Zhen, Yinchun Huang, Penghu Wei, Yongzhi Shan

**Affiliations:** ^1^ Department of Neurosurgery XuanWu Hospital, Capital Medical University Beijing China; ^2^ International Neuroscience Institute (China‐INI) Beijing China; ^3^ Clinical Research Center for Epilepsy Capital Medical University Beijing China

**Keywords:** corpus callosotomy, drop attacks, drug‐resistant epilepsy, seizure freedom

## Abstract

**Background:**

Corpus callosotomy (CC) is appropriate for patients with seizures of a bilateral or diffuse origin, or those with seizures of a unilateral origin with rapid spread to the contralateral cerebral hemisphere. The efficiency of CC in patients with drug‐resistant epilepsy is a long‐term concern because most articles reporting the surgical results of CC arise from small case series, and the durations of follow‐up vary.

**Methods:**

PubMed, Embase, Cochrane Library, and Web of Science were searched to identify papers published before November 8, 2021. The systematic review was completed following PRISMA guidelines. Outcomes were analyzed by meta‐analysis of the proportions.

**Results:**

A total of 1644 patients with drug‐resistant epilepsy (49 retrospective or prospective case series studies) underwent CC, and the follow‐up time of all patients was at least 1 year. The rate of complete seizure freedom (SF) was 12.38% (95% confidence interval [CI], 8.17%–17.21%). Meanwhile, the rate of complete SF from drop attacks was 61.86% (95% CI, 51.87%–71.41%). The rates of complete SF after total corpus callosotomy (TCC) and anterior corpus callosotomy (ACC) were 11.41% (95% CI, 5.33%–18.91%) and 6.75% (95% CI, 2.76%–11.85%), respectively. Additionally, the rate of complete SF from drop attacks after TCC was significantly higher than that after ACC (71.52%, 95% CI, 54.22%–86.35% vs. 57.11%, 95% CI, 42.17%–71.49%). The quality of evidence for the three outcomes by GRADE assessment was low to moderate.

**Conclusion:**

There was no significant difference in the rate of complete SF between TCC and ACC. TCC had a significantly higher rate of complete SF from drop attacks than did ACC. Furthermore, CC for the treatment of drug‐resistant epilepsy remains an important problem for further investigation because there are no universally accepted standardized guidelines for the extent of CC and its benefit to patients. In future research, we will focus on this issue.

## Graphical abstract

There was no significant difference in the rate of complete SF between TCC and ACC. TCC had a significantly higher rate of complete SF from drop attacks than the ACC did. Furthermore, CC for the treatment of RE is still an important problem for further discussion because there are still no universally accepted standardized guidelines for the extent of CC and its benefit to patients. In future research, we will focus on this issue.

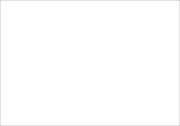

## 1 INTRODUCTION

Epilepsy is a common disease of the nervous system with an incidence of 0.6%−1.1% of the general population. Seizure freedom (SF) can be achieved with appropriate medication in most patients (Brodie, 2013; Duncan et al., 2006). However, more than a third of patients continue to have seizures while taking antiseizure medications. Surgery is an effective treatment method for some patients with drug‐resistant epilepsy (DRE). Surgical resection of epileptogenic foci is suitable for patients with clear lesions and limited epileptic discharges (Englot & Chang, 2014; Tellez‐Zenteno et al., 2005). However, for patients with seizures of a bilateral or diffuse origin, or those with seizures of a unilateral origin with rapid spread to the contralateral cerebral hemisphere, corpus callosotomy (CC) is appropriate. CC is considered a palliative surgical treatment for patients with DRE, particularly those with multifocal or generalized epilepsy (Asadi‐Pooya et al., 2008; Otsuki et al., 2015), as focal resection is impractical in these patients. CC is performed based on the pathophysiological hypothesis that the corpus callosum is the main route for transmission of epileptic discharges between the two hemispheres of the brain. The mechanism of CC involves severing the fibers connecting the hemispheres of the brain, preventing interhemispheric spread of epileptic discharges (Duc Lien et al., 2020; Passamonti et al., 2014; You et al., 2008).

There have been several systematic reviews and meta‐analyses of surgeries for DRE, but only two articles have focused on CC (Chan et al., 2018; Graham et al., 2016), while others have compared CC to other treatments, such as vagus nerve stimulation (VNS) and ketogenic diet (Rolston et al., 2015; Sharawat et al., 2021; Ye et al., 2021). Most reports exploring the surgical results of CC in patients with DRE arise from small case series. The rate of complete SF (freedom from all seizure types) remains unclear. While differences exist among studies because of the lack of universally accepted standardized outcome measures and guidelines, CC is well‐documented to be an effective and safe treatment (Cendes et al., 1993; Geoffroy et al., 1983; Jalilian et al., 2010; Kwan et al., 2000). However, among these studies, the shortest follow‐up times varied significantly. The recurrence rate of epilepsy after surgery is known to increase over time. We conducted a systematic review and quantitative analysis of many papers to analyze these surgical outcomes. We focused on the overall rates of complete SF and freedom from drop attacks after CC, especially those more than 1 year after CC.

## 2 MATERIALS AND METHODS

**FIGURE 1:**
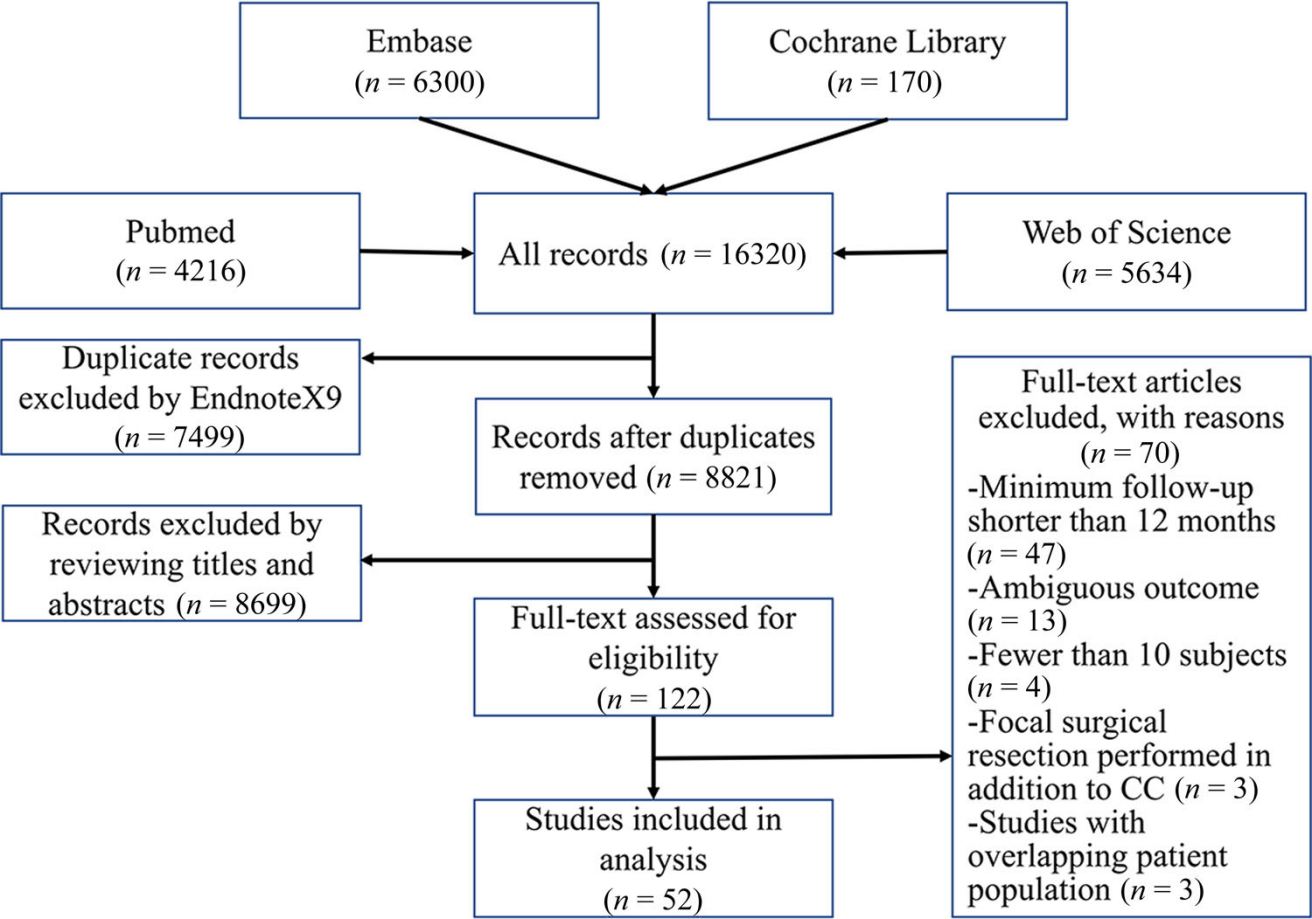
Flowchart of search results according to the PRISMA. PRISMA, Preferred Reporting Items for Systematic Reviews and Meta‐Analyses.

This study was carried out in accordance with the PRISMA guidelines (Moher et al., 2009; Wu, Song, et al., 2021). A PRISMA flow diagram is shown in Figure 1.

### 2.1 Literature search

The last literature search was performed on December 12, 2022. We searched the following four databases: PubMed, Embase, Cochrane Library, and Web of Science. We searched for articles reporting the surgical results of CC for patients with DRE, focusing on the rate of complete SF, especially more than 1 year after CC. Keyword searches were performed according to the search strategy. The inclusion criteria were as follows: (1) original research articles; (2) the surgical results of CC or patients with DRE were reported, focusing on the rate of complete SF; (3) the follow‐up time of all patients was at least 1 year; (4) the article included at least 10 human patients; and (5) the study was written in English; there were no additional requirements for publication dates. The exclusion criteria were as follows: (1) case reports, reviews, editorials, or series including results from fewer than 10 patients; (2) absent or ambiguous outcome data; (3) the minimum follow‐up was shorter than 1 year; (4) focal surgical resection was performed in addition to CC; (5) all reported cases were clearly defined as patients with recurrent seizures after VNS; (6) patients who only underwent laser interstitial thermal therapy; and (7) articles dealing with cadaver research or in a non‐English language.

### 2.2 Bias and quality assessment

Part of the Methodological Index for Non‐Randomized Studies (Slim et al., 2003) was used to assess the quality of the studies. The items were scored as 0 (not reported), 1 (reported but inadequate), or 2 (reported and adequate). The maximum score for noncomparative studies was 16. A higher score indicated a higher quality study. When discrepancies arose, the papers were re‐examined by the third author. Studies with scores of 11 or greater were considered high quality (Slim et al., 2003; Wu, Li, et al., 2021). The quality assessment was independently performed by two researchers. The third author re‐examined the papers when discrepancies arose. We used the Grading of Recommendations Assessment, Development and Evaluation (GRADE) framework to assess the certainty of evidence (Guyatt et al., 2008). Egger's test and a funnel plot were used to assess publication bias.

### 2.3 Extraction of data and meta‐analysis

Data extraction was completed independently by two researchers. The primary outcome was the overall seizure outcome at the follow‐up at least 1 year after CC; outcomes were extracted and classified into “complete SF” and “incomplete SF.” The extent of CC was dichotomized as total or partial (anterior and posterior). Subgroup analysis, including total corpus callosotomy (TCC) versus anterior corpus callosotomy (ACC), was also performed. If seizure outcomes were not satisfactory in some patients who underwent ACC, they might proceed to perform a further section of the corpus callosum. In addition, the drop attack outcome was also extracted and classified into “drop attack‐free” and “not drop attack‐free.” Only clearly defined “drop attack” seizures were included in our study. We attempted to obtain any missing or ambiguous information by contacting the corresponding authors of the studies. Meta‐analysis was performed using R‐5.0.3 (R Foundation for Statistical Computing, Vienna, Austria). The meta‐package (version 5.1‐1) was used to compute the rates of SF with 95% confidence interval (95% CI). The rates of SF presented for each article were double‐arcsine transformed (Freeman & Tukey, 1950; Wang, 2018; Wu, Li, et al., 2021; Wu, Song, et al., 2021). The Q test and *I* ^2^ statistic were used to estimate statistical heterogeneity among different studies. The rate of SF was computed using a fixed‐effects model (*I* ^2^ < 50% and *p* > .1), and a random‐effects model was used to compute the rate of SF (*I* ^2^ > 50% and *p* < .1) (Higgins & Thompson, 2002; Higgins et al., 2003; Sedgwick, 2015). Meta‐regression and sensitivity analysis were conducted to explore the potential heterogeneity. Meta‐regression was used to describe a linear relationship between variables and the effect size, and we conducted it to explore the potential effect on the rate of complete SF based on the study publication year.

## 3 RESULTS

### 3.1 Literature review

Details of search strategies are available in Supporting Information S1. In total, we identified 16,320 articles from medical databases. EndnoteX9 software was used to automatically screen and exclude duplicate records. A total of 122 full texts of articles were reviewed. The flow diagram is shown in Figure 1. Finally, 52 retrospective or prospective case series studies (Andersen et al., 1996; Asadi‐Pooya et al., 2013; Baba et al., 2018; Carmant et al., 1998; Cendes et al., 1993; Cohen & Author et al., 1991; Cukiert et al., 2006, 2009, 2013; Duc Lien et al., 2020; Fandiño‐Franky et al., 2000; Ferrand‐Sorbets et al., 2022; Frigeri et al., 2021; Gates et al., 1987; Honda et al., 2021; Iwasaki et al., 2016; Kagawa et al., 2021; Kanai et al., 2020; Kawai et al., 2004; Kim et al., 2004; Kwan et al., 2001, 2006; Liang et al., 2010, 2014, 2015; Lin & Kwan, 2012; Maehara & Shimizu, 2001; Makari et al., 1989; Mamelak et al., 1993; Na et al., 2022; Nordgren et al., 1991; Oguni et al., 1991; Otsuki et al., 2015; Paglioli et al., 2016; Passamonti et al., 2014; Ping et al., 2009; Rathore et al., 2007; Rossi et al., 1996; Sadashiva et al., 2023; Sakas & Phillips, 1996; Shim et al., 2008; Shimizu, 2005; Spencer et al., 1991; Stigsdotter‐Broman et al., 2014; Sunaga et al., 2009; Tanriverdi et al., 2009; Thohar Arifin et al., 2020; Turanli et al., 2006; Ueda et al., 2019; Ukishiro et al., 2022; Yang et al., 2014; You et al., 2008) encompassing 1827 patients were included. The demographic characteristics are shown in Supporting Information S2.

### 3.2 Quality assessment

In the “case series” group, 34 studies were graded as “high quality” and 18 were graded as “low quality.” The prospective randomized study was graded as “low risk (high quality).” Detailed information is provided in Supporting Information S3. There was complete agreement between the two reviewers for 47 studies and 34 were rated as “high quality.” Discrepancies arose for five studies, all of which were rated as “low quality,” and the final determination was made by the third author, who made the decision to rate them as “low quality.” The disagreements arose for the following reasons: (1) poorly defined patient inclusion criteria, (2) unclear description of the inclusion of consecutive patients and prospective collection of data, and (3) a lack of demographic and clinical data.

### 3.3 The rate of complete SF

**FIGURE 2:**
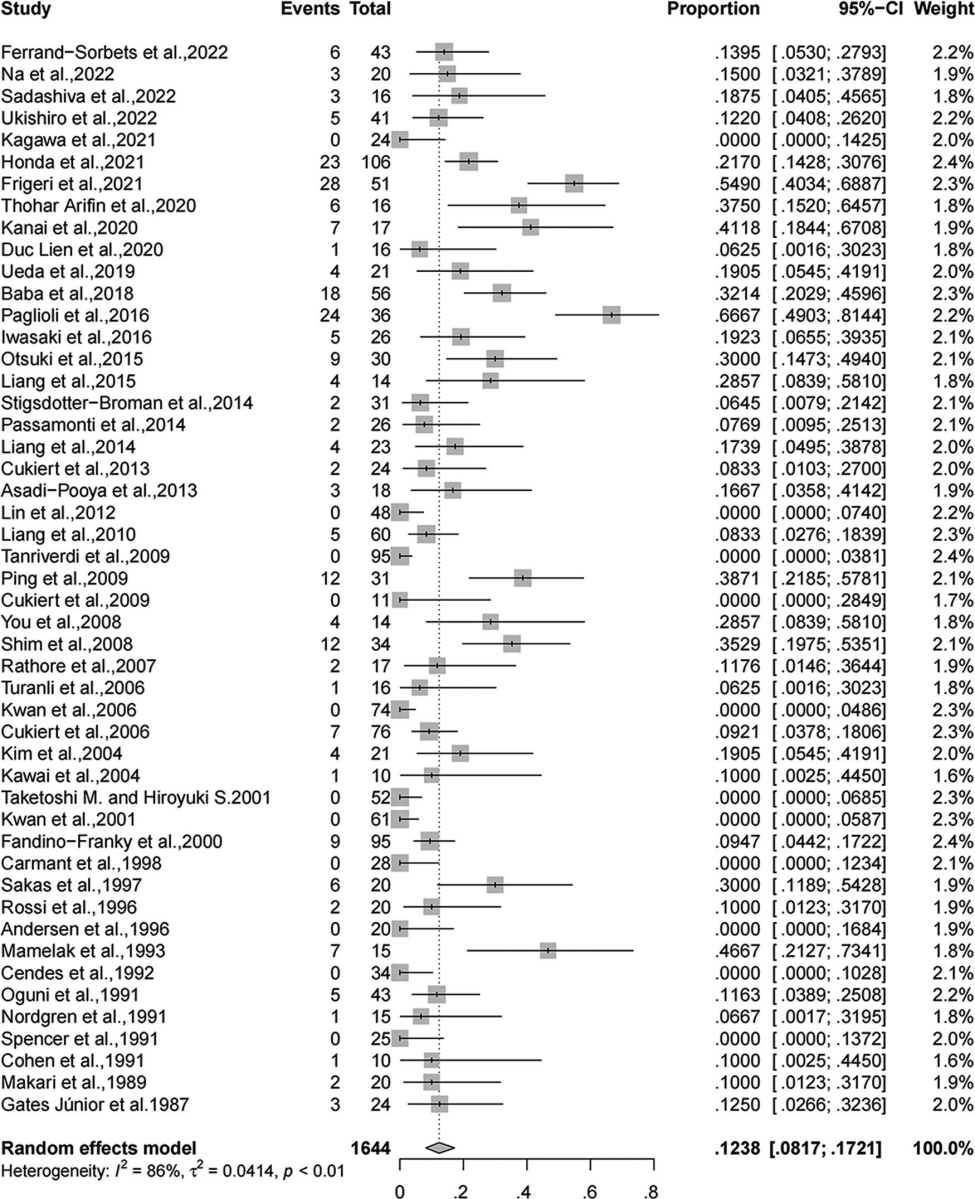
Forest plot of meta‐analysis for the rate of complete SF. SF, seizure freedom.

**FIGURE 3:**
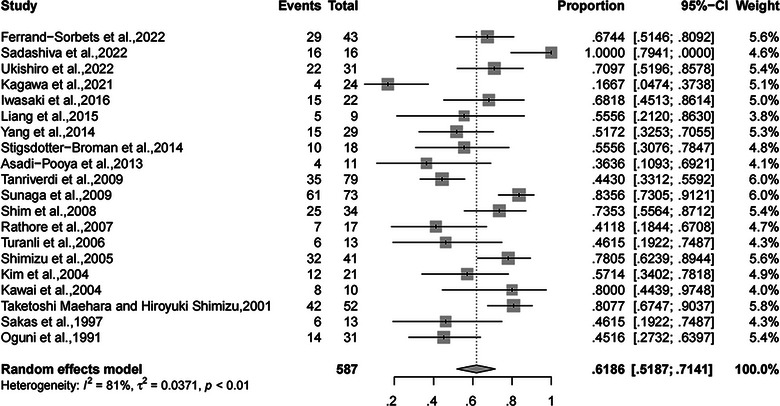
Forest plot of meta‐analysis for the rate of complete SF from drop attacks. SF, seizure freedom.

A total of 1644 patients with DRE (49 retrospective or prospective case series studies) underwent CC surgery, and the follow‐up time of all patients was at least 1 year. This is because three studies (Shimizu, 2005; Sunaga et al., 2009; Yang et al., 2014) did not explicitly report the number of patients with complete SF. The rate of complete SF was 12.38% (95% confidence interval [CI], 8.17%−17.21%, random‐effects; Figure 2), with significant heterogeneity (*I* ^2^ = 86%; 95% CI, 82.6%−89.1%; *p* < .01). Meanwhile, the rate of complete SF from drop attacks was 61.86% (95% CI, 51.87%−71.41%, random‐effects; Figure 3), with relatively high heterogeneity (*I* ^2^ = 81%, 95% CI, 71.5%−87.3%; *p* < .01).

### 3.4 Subgroup analysis

**FIGURE 4:**
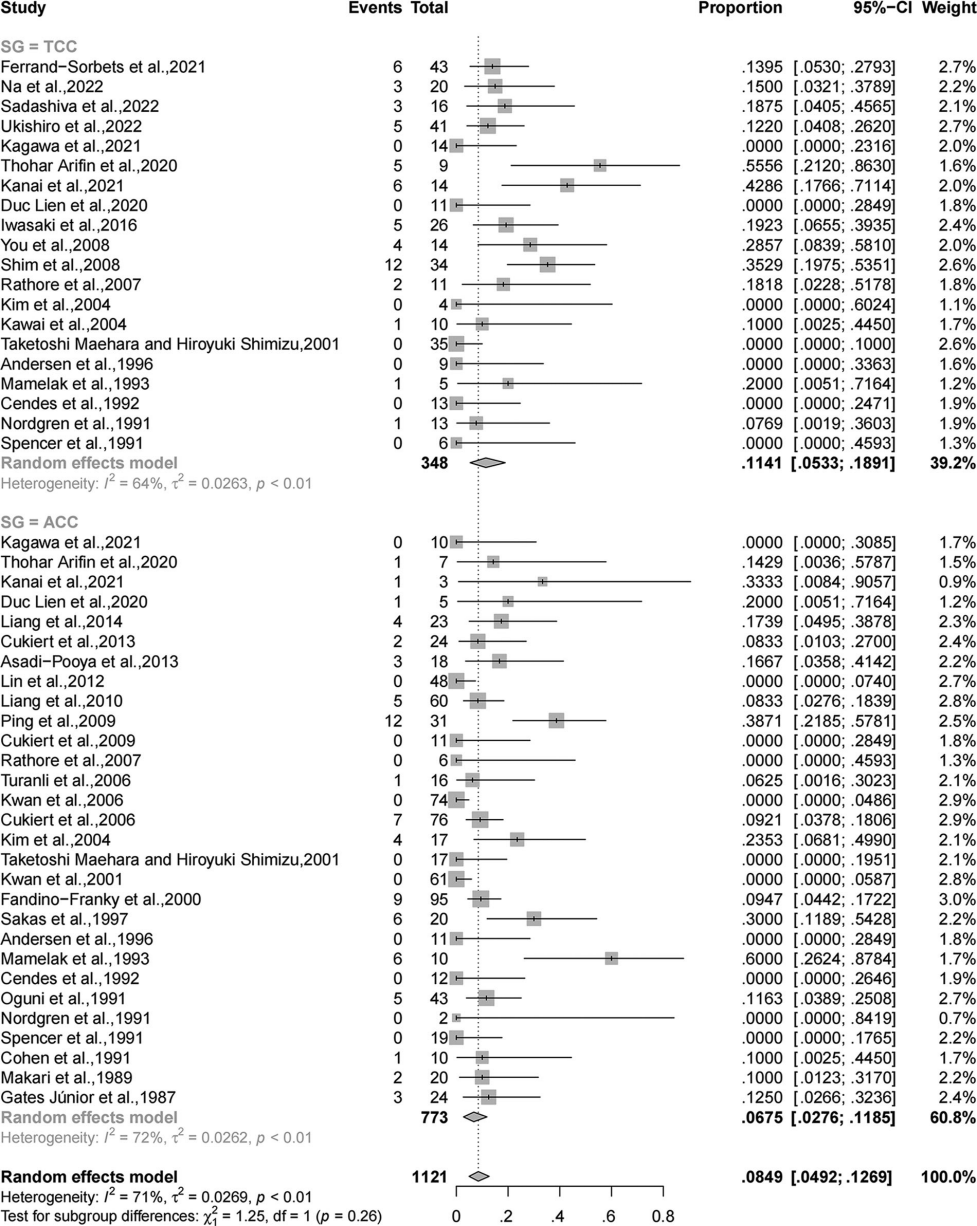
Forest plot of subgroup analysis for the rates of complete SF after TCC and ACC. SF, seizure freedom; TCC, total corpus callosotomy; ACC, anterior corpus callosotomy.

**FIGURE 5:**
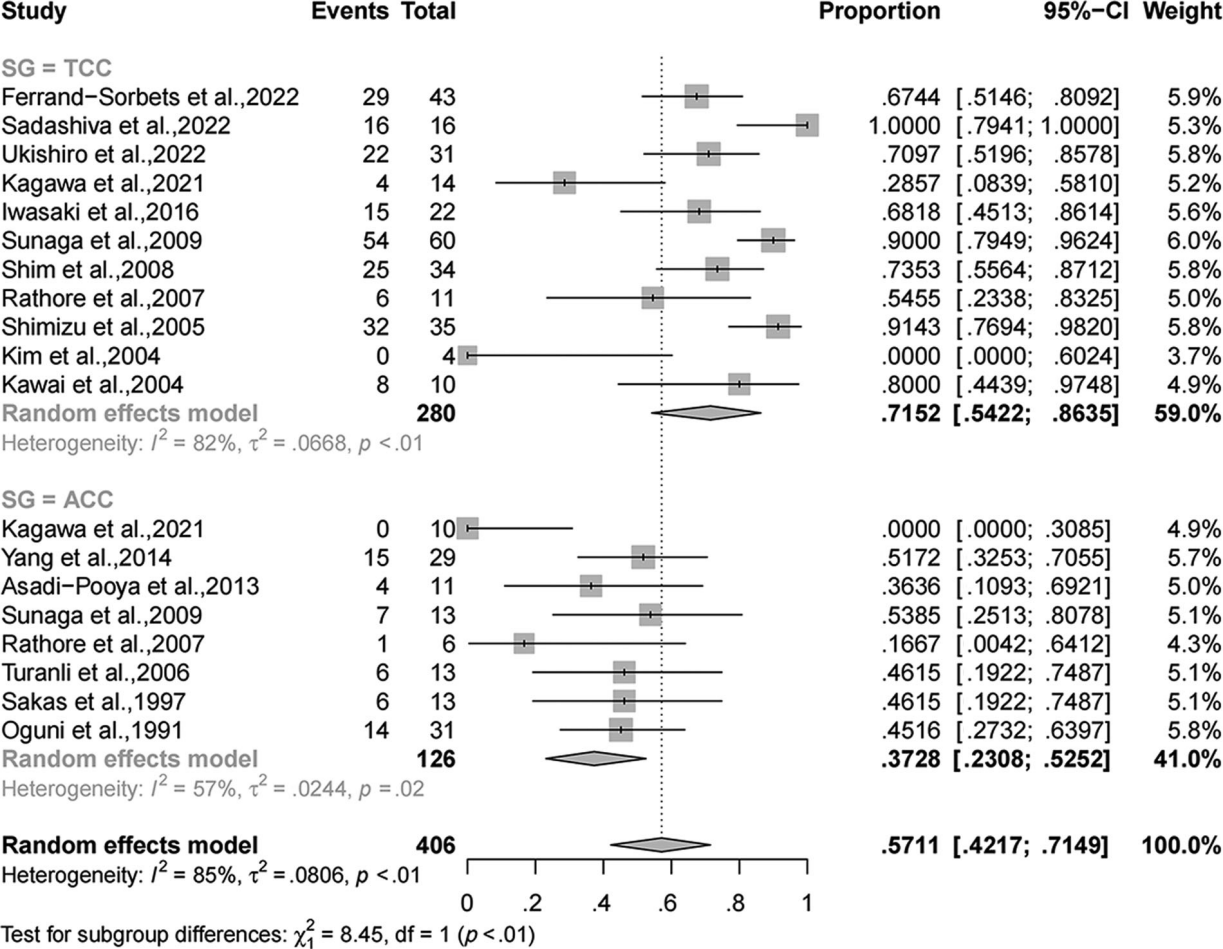
Forest plot of subgroup analysis for the rates of complete SF from drop attacks after TCC and ACC. SF, seizure freedom; TCC, total corpus callosotomy; ACC, anterior corpus callosotomy.

A subgroup analysis was performed to reveal the extent of CC (total and anterior) that led to a different rate of complete SF. The rates of complete SF after TCC and ACC were 11.41% (95% CI, 5.33%−18.91%) and 6.75% (95% CI, 2.76%−11.85%), respectively, without a significant difference (*p* = .26, random‐effects; Figure 4). Another subgroup analysis was performed to compare rates of complete SF from drop attacks between the different extents of CC (total and anterior) (*p* < .01, random‐effects; Figure 5). In the random‐effects model, the rate of complete SF from drop attacks after TCC was significantly higher than that after ACC (71.52%, 95% CI, 54.22%−86.35% vs. 57.11%, 95% CI, 42.17%−71.49%).

### 3.5 Acute disconnection syndrome

In a formal meta‐analysis of available data, the rate of acute disconnection syndrome after CC was 11.99% (95% CI, 3.35%−24.08%), with significant heterogeneity (*I* ^2^ = 96%; 95% CI, 94.9%−96.7%; *p* < .01; Supporting Information S4).

### 3.6 Exploration of heterogeneity

**FIGURE 6:**
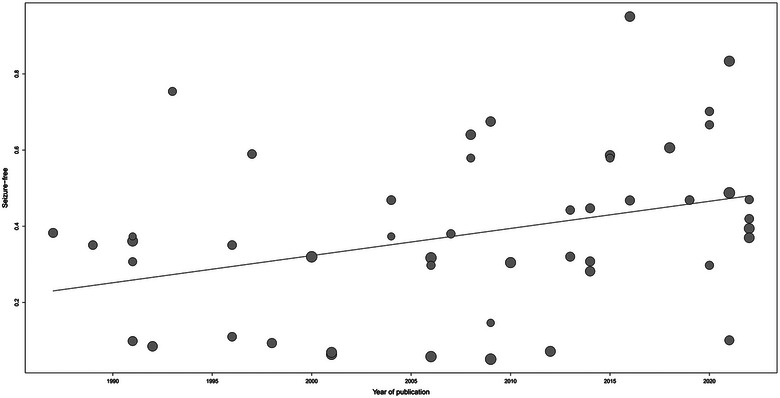
Meta‐regression plot showing that the rate of complete SF increased with the year of publication. SF, seizure freedom.

**FIGURE 7:**
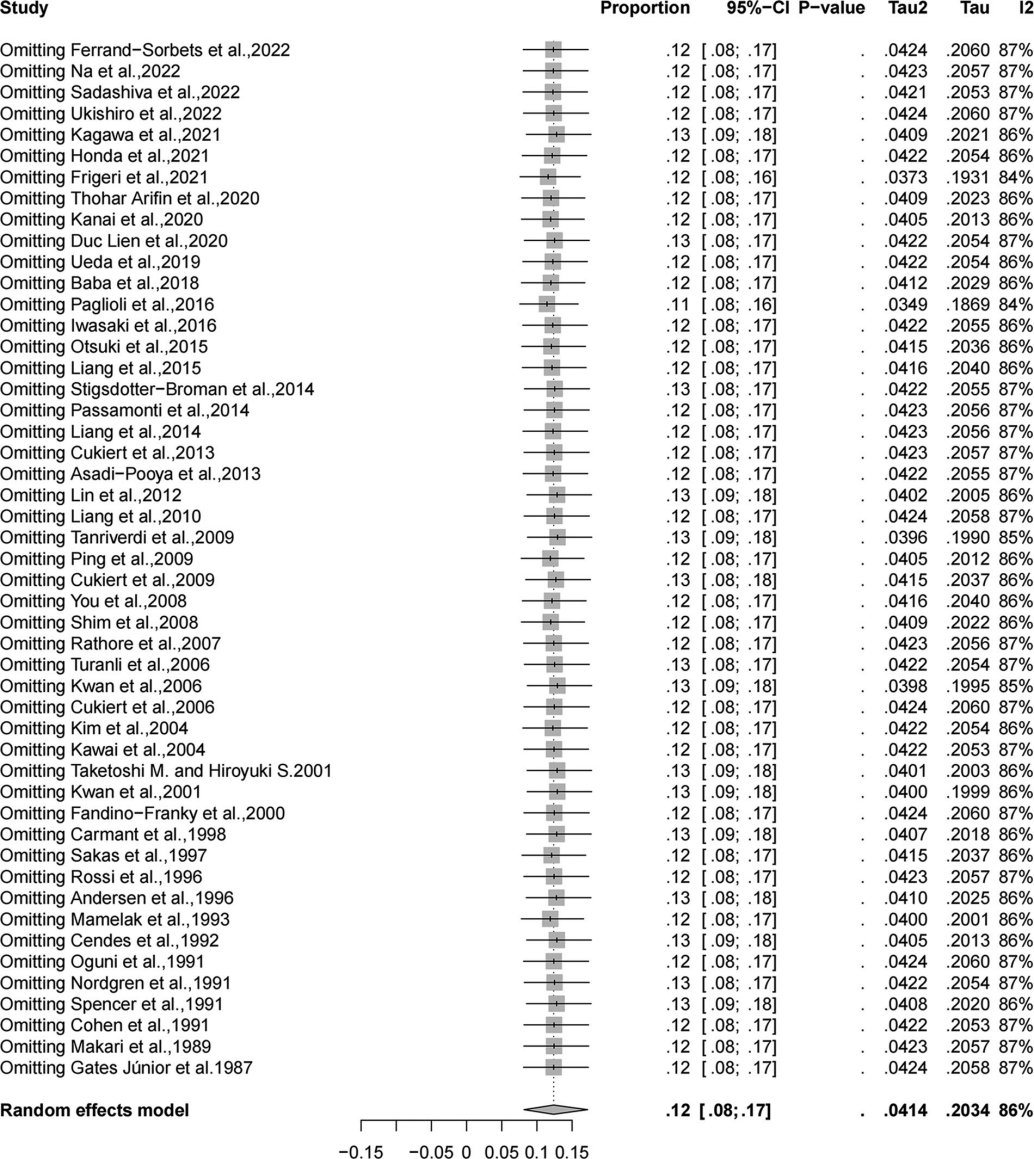
Results of sensitivity analysis for the rates of complete SF. The figure displays the rate of complete SF obtained from combined calculations involving the remaining studies, after each study has been sequentially eliminated. SF, seizure freedom.

According to the univariate meta‐regression analysis, the rate of complete SF increased with the year of publication (*p* = .01; Figure 6). The results also suggested that publication year might be a source of high heterogeneity. Sensitivity analysis indicated that none of the studies had a strong effect on the rate of complete SF (Figure 7). The absence of publication bias was confirmed using Egger's weighted regression test (*p* = .12) and visual inspection of funnel plot symmetry (Supporting Information S5). The quality of evidence for the three outcomes was low to moderate by the GRADE assessment. The certainty of evidence was moderate to low by the GRADE assessment (Supporting Information S6).

## 4 DISCUSSION

The efficiency of patients with DRE after CC is a long‐term concern because most articles reporting surgical results of CC arise from small case series with varying durations of follow‐up. Our results showed that the rate of complete SF more than 1 year after CC was 12.38%, and the rate of complete SF from drop attacks was 61.86%. Meanwhile, the rate of complete SF from drop attacks after TCC was significantly higher than that after ACC (71.52% vs. 57.11%). Currently, the palliative surgical treatment for DRE mainly includes CC and VNS. Ye et al. (2021) pointed out that CC is superior to VNS for the management of atonic seizures in the pediatric population. A meta‐analysis comparing the efficacy of different surgical treatments for controlling Lennox–Gastaut syndrome found that the rate of seizure reduction was significantly higher than VNS (74.1% vs. 54.6%, *p* < .001) (Thirunavu et al., 2021). Rolston et al. (2015) believed that although both CC and VNS are effective in the treatment of RE, CC seems to have better results in terms of achieving complete SF than VNS, especially for patients with drop attacks. Another emerging treatment is responsive neurostimulation, but no large‐scale series have been reported for this treatment. Bercu et al. (2020) reported six patients with DRE who were treated with responsive neurostimulation. They believed that this procedure was safe and had a good result in terms of decreasing seizure frequency. Our results also demonstrate the long‐term effectiveness of CC, especially in patients with drop attacks. However, to confirm this point, it is necessary to further clarify the advantages and disadvantages of CC in terms of short‐term trauma and long‐term quality of life for patients with DRE, compared with other palliative treatments.

In most centers, CCs can be classified into TCC, ACC, and posterior corpus callosotomy (PCC) by sectioning variable extents. Some believe that the ACC could allow patients to preserve more neurological functions by leaving the splenium intact (Liang et al., 2010; Meguins et al., 2015; Rahimi et al., 2007). However, other studies have supported the use of TCC for achieving more effective clinical results (Bower et al., 2013; Meguins et al., 2015). This meta‐analysis included 52 studies from different countries from 1987 to 2022, with a rate of complete SF ranging from 0% to 66.67%. One meta‐analysis reported SF to be less than 18.8% (Chan et al., 2018), and there are many studies on prognosis after CC (Andersen et al., 1996; Cukiert et al., 2006, 2013; Honda et al., 2021; Kwan et al., 2006; Liang et al., 2010; Ping et al., 2009; Thohar Arifin et al., 2020; Ueda et al., 2019; Yang et al., 2014). However, outcomes of complete SF surgery vary greatly.

### 4.1 History and evolution of CC

Dandy presented the first partial resection of the corpus callosum in 1936. Postoperatively, patients did not show significant neurological deficits. Van Wagenen and Herren published a study (Van Wagenem & Herren, 1940) on treatments for epilepsy, which included 10 patients; this was the first description of CC as a surgical treatment for patients with DRE. This hypothesis has long been the pathophysiological basis of CC for the surgical treatment of DRE. As a palliative surgical treatment for patients with DRE exceptionally characterized by generalized or multifocal epilepsy, CC seems to be a suitable option for reducing the burden of different DRE conditions and improving quality of life (Chan et al., 2018; Duc Lien et al., 2020; Graham et al., 2016). In 1970, Luessenhop et al. reported that CC is more widely used than hemispherectomy in the surgical treatment of DRE because it can be performed in both children and adults with severe hemiplegia and hemianopia. CC is considered safe in both children and adults (Luessenhop, 1970; Luessenhop & Fenichel, 1970). Increasingly, CC has emerged as an alternative to hemispherectomy. Sperry and his colleagues found that anterior commissure and TCC, carried out in a single operation, almost completely controlled generalized convulsions for long periods of time. Additionally, Sperry believed verbal intelligence, numeracy, established motor coordination, personality, and temperament were not damaged by commissurotomy (Blume, 1984; Gordon et al., 1971; Sperry, 1974). Since the 1990s, CC procedures have gradually become standardized. Meanwhile, the development goal of CC is to obtain the best results with minimal injury (Devinsky & Laff, 2003; Smyth et al., 2017).

### 4.2 Extent of CC

Traditionally, the ACC includes different extents of disconnection. In other words, a disconnection between the bilateral frontal lobes would be achieved in all patients. ACC is the most commonly used approach for controlling drop attacks. However, some scholars are skeptical, and argue that ACC does not yield satisfactory results. They believe the rate of complete SF or control of drop attacks is usually lower than that of TCC. Similarly, our results showed that TCC was associated with a significantly higher rate of complete SF from drop attacks than ACC. However, we did not find a significant difference between the rates of complete SF for TCC and ACC. The significantly better results for TCC were due to the disconnection of fibers from the premotor and primary motor cortex, which crossed over the mid to posterior half, rather than the anterior half of the corpus callosum. This concept is supported by anatomy and imaging data (Aboitiz et al., 1992; Naets et al., 2015; Paglioli et al., 2016; Zarei et al., 2006). Zarei et al. (2006) reported a tractography study in which fibers originating from the premotor, supplementary sensorimotor area, and primary motor cortices crossed the isthmus of the corpus callosum up to the borders of the splenium. This supports our finding that TCC was associated with a significantly higher rate of complete SF from drop attacks than was ACC. Therefore, PCC is increasingly being applied clinically. Paglioli et al. (2016) presented a clinical series that included 36 patients with drop attacks. After follow‐up for at least 4 years, 30 patients achieved either complete or 90% control of the drop attacks. They proposed that PCC is safe, is effective, and achieves better results in controlling drop attacks compared to other treatments, similar to previous clinical series investigating TCC.

### 4.3 Disconnection syndrome

In most studies, only acute disconnection syndrome has been mentioned. Patients who developed acute disconnection syndrome almost completely recovered within a few months. In our study, the acute disconnection syndrome incidence rate after surgery was 11.99%. However, as acute disconnection syndrome is difficult to evaluate in severely injured patients, these data should be interpreted with caution. Typically, TCC is considered to lead to more complications than ACC, including disconnection syndrome (Harbaugh et al., 1983; Meguins et al., 2015). Turanli et al. (2006) believed that CC had better effects in children than in adults because of the continuous development of their brains. Outcomes of TCC are much better than those of partial CC for the control and prevention of epileptic recurrence. However, other studies have suggested that patients have better outcomes after TCC (Cukiert et al., 2006; Shim et al., 2008). A systematic review and meta‐analysis of 33 studies divided them into two subgroups (TCC vs. ACC). TCC was found to be better than ACC in reducing the frequency of seizures in pediatric patients (Meguins et al., 2015). Bower et al. (2013) and Kasasbeh et al. (2014) did not observe any significant differences between the TCC and ACC subgroups in terms of blood loss, frequency of worsened seizures or new seizure types, and disconnection syndrome. In many respects, insufficient data are available for meta‐analyses of different complications.

### 4.4 Limitations

This study had several limitations. (1) All results in this article showed significant heterogeneity. This could be explained by several factors, including surgical experience, patient characteristics (e.g., we included all outcomes of both children and adults), and the lack of universally accepted standardized outcome measures and guidelines for CC. (2) Since most patients reported in the literature included both children and adults, it was difficult to separate outcomes between children and adults in our article. (3) The studies were written in English. (4) Most of the included studies were retrospective. (5) It is difficult to perform further analyses due to the unavailability of data. (6) The meta‐analysis was not registered and protocol was not prepared; therefore, there is a potential risk of bias. Nevertheless, this study was strictly carried out in accordance with the PRISMA guidelines.

## 5 CONCLUSION

Our results showed that the rate of complete SF was 12.38%, and the rate of freedom from drop attacks was 61.86%. There were no significant differences in the rates of complete SF between TCC and ACC. TCC had a significantly higher rate of complete SF from drop attacks than did ACC. Furthermore, CC for the treatment of DRE remains an important problem for further investigation, because there are no universally accepted standardized guidelines for the extent of CC and its benefits to patients. In future research, we will focus on this issue.

## AUTHOR CONTRIBUTIONS

*Literature search*: Xiaolong Wu, Siqi Ou, and Huaqiang Zhang. *Extraction of data*: Xiaolong Wu, Yuhang Zhen, Huaqiang Zhang, and Yinchun Huang. *Quality assessment*: Xiaolong Wu, Siqi Ou, and Huaqiang Zhang. *Statistical analysis and writing original draft*: Xiaolong Wu. *Review and editing*: Yongzhi Shan and Penghu Wei.

## CONFLICT OF INTEREST STATEMENT

The authors declare no conflicts of interest.

### PEER REVIEW

The peer review history for this article is available at https://publons.com/publon/10.1002/brb3.2964.

## Supporting information

Supplementary Materials 1

Supplementary Materials 2. Demographic characteristics

Supplementary Materials 3. The quality of the case series studies was assessed by part of the METHODOLOGICAL INDEX FOR NON‐RANDOMIZED STUDIES (MINORS) Scale

Supplementary Materials 4

Supplementary Materials 5

Supplementary Materials 6. GRADE Assessment

## Data Availability

The authors declare the data were collected from publicly available sources. Data and code can be provided upon reasonable request.
