# Supplementary material for: Long‐term follow‐up seizure outcomes after corpus callosotomy: A systematic review with meta‐analysis
Source: Brain Behav. 2023 Mar 16;13(4):e2964. doi: 10.1002/brb3.2964 (PMC10097058; doi:10.1002/brb3.2964)
Supplement: Supplementary file 1 — Supplementary Materials 1 [file BRB3-13-e2964-s003.docx]

**Supplementary Materials 1**

2022.12.12Pubmed

|  | Search strategy | Results |
| --- | --- | --- |
| #1 | ((((corpus callosum[MeSH Terms]) OR (corpus callosum[Title/Abstract])) OR (callosum[Title/Abstract])) OR (splenium[Title/Abstract])) OR (callosal[Title/Abstract]) | 24102 |
| #2 | (((((surgery[Title/Abstract]) OR (surgical[Title/Abstract])) OR (operation[Title/Abstract])) OR (resection[Title/Abstract])) OR (section[Title/Abstract])) OR (treatment[Title/Abstract]) | 6867853 |
| #3 | #1 AND #2 | 4101 |
| #4 | (((corpus callosotomy[Title/Abstract]) OR (callosotomy[Title/Abstract])) OR (callosal disconnection syndrome[Title/Abstract])) OR (callosal disconnection[Title/Abstract]) | 1020 |
| #5 | #3 OR #4 | 4680 |
| #6 | #5 AND (english[Filter]) | 4216 |

2022.12.12Embase

|  | Search strategy | Results |
| --- | --- | --- |
| #1 | 'corpus callosum':ab,ti OR callosum:ab,ti OR splenium:ab,ti OR callosal:ab,ti | 29570 |
| #2 | surgery:ab,ti OR surgical:ab,ti OR operation:ab,ti OR resection:ab,ti OR section:ab,ti OR treatment:ab,ti | 9419336 |
| #3 | #1 AND #2 | 5764 |
| #4 | 'corpus callosotomy':ab,ti OR callosotomy:ab,ti OR 'callosal disconnection syndrome':ab,ti OR 'callosal disconnection':ab,ti | 1440 |
| #5 | #3 OR #4 | 6898 |
| #6 | #5 AND [english]/lim | 6300 |

2022.12.12Cochrane library

|  | Search strategy | Results |
| --- | --- | --- |
| #1 | (surgery):ti,ab,kw OR (surgical):ti,ab,kw OR (operation):ti,ab,kw OR (resection):ti,ab,kw OR (treatment):ti,ab,kw OR (section):ti,ab,kw (Word variations have been searched) | 1091138 |
| #2 | MeSH descriptor: [Corpus Callosum] explode all trees | 69 |
| #3 | (corpus callosum):ti,ab,kw OR (callosum):ti,ab,kw OR (splenium):ti,ab,kw OR (callosal):ti,ab,kw | 290 |
| #4 | (#2 OR #3) AND #1 | 140 |
| #5 | (corpus callosotomy):ti,ab,kw OR (callosotomy):ti,ab,kw OR (Callosal Disconnection Syndrome):ti,ab,kw OR (Disconnection Syndrome):ti,ab,kw | 41 |
| #6 | #4 OR #5 | 170 |

2022.12.12Web of science

|  | Search strategy | Results |
| --- | --- | --- |
| #1 | (((((((TI=(corpus callosum)) OR AB=(corpus callosum)) OR TI=(callosum)) OR AB=(callosum)) OR TI=(splenium)) OR AB=(splenium)) OR TI=(callosal)) OR AB=(callosal) | 23470 |
| #2 | (((((((((((TI=(surgery)) OR AB=(surgery)) OR TI=(surgical)) OR AB=(surgical)) OR TI=(operation)) OR AB=(operation)) OR TI=(resection)) OR AB=(resection)) OR TI=(treatment)) OR AB=(treatment)) OR TI=(section)) OR AB=(section) | 21850085 |
| #3 | #1 AND #2 | 4605 |
| #4 | (((((((TI=(corpus callosotomy)) OR AB=(corpus callosotomy)) OR TI=(callosotomy)) OR AB=(callosotomy)) OR TI=(Callosal Disconnection Syndrome)) OR AB=(Callosal Disconnection Syndrome)) OR TI=(Callosal Disconnection)) OR AB=(Callosal Disconnection) | 1306 |
| #5 | (#3) OR #4 | 5634 |
