# Supplementary material for: Long‐term follow‐up seizure outcomes after corpus callosotomy: A systematic review with meta‐analysis
Source: Brain Behav. 2023 Mar 16;13(4):e2964. doi: 10.1002/brb3.2964 (PMC10097058; doi:10.1002/brb3.2964)
Supplement: Supplementary file 2 — Supplementary Materials 2. Demographic characteristics [file BRB3-13-e2964-s006.docx]

**Supplementary Materials 2. Demographic characteristics**

| Study | Study design | Country | Sample size | Extent | | | Drop attacks | | | Sex (M/F) | | | Age at surgery（years） | | | Follow-up（years） | | | | complete control（Seizure freedom） | | | evaluation criterion | Control of drop attacks | | | acute disconnection syndrome | | |
| --- | --- | --- | --- | --- | --- | --- | --- | --- | --- | --- | --- | --- | --- | --- | --- | --- | --- | --- | --- | --- | --- | --- | --- | --- | --- | --- | --- | --- | --- |
|  |  |  |  | TCC | ACC | PCC | TCC | ACC | PCC | TCC | ACC | PCC | TCC | ACC | PCC | TCC | ACC | | PCC | TCC | ACC | PCC |  | TCC | ACC | PCC | TCC | ACC | PCC |
| Ferrand-Sorbets et al.,2022 | retrospective cohort | France | 43 | 43 | / | / | 43 | / | / | 28/15 | / | / | 6.22 | / | / | 1 | / | | / | 6 | / | / | self-defined | 29 | / | / | 1 | / | / |
| Na et al.,2022 | retrospective cohort | Korea | 20 | 20 | / | / | / | / | / | 15/5 | / | / | 6.68 | / | / | 1 | / | | / | 3 | / | / | NA | / | / | / | / | / | / |
| Sadashiva et al.,2022 | retrospective cohort | India | 16 | 16 | / | / | 16 | / | / | NA | / | / | NA | / | / | a minimum of 1year | | / | / | 3 | / | / | Engel class as well as ILAE outcome scales | 16 | / | / | NA | / | / |
| Ukishiro et al.,2022 | retrospective cohort | Japan | 41 | 41 | / | / | 31 | / | / | 19/22 | / | / | 10.6 | / | / | at least 1 year | | / | / | 5 | / | / | criteria of Williamson | 22 | / | / | 0 | / | / |
| Kagawa et al.,2021 | retrospective cohort | Japan | 24 | 14 | 10 | 0 | 14 | 10 | 0 | 11NA3 | 7NA3 | 0 | 16^#^ | 22.5^#^ | NA | a minimum follow-up of one year | | | NA | 0 | 0 | NA | Rathore’s class | NA | NA | NA | 1 | 1 | NA |
| Honda et al.,2021 | retrospective cohort | Japan | 106 | 101 | 5 | 0 | NA | NA | NA | 55/51 | | 0 | 2.525 | | NA | 1 | | | NA | 23 | | NA | NA | NA | NA | NA | NA | NA | NA |
| Frigeri et al.,2021 | prospective cohort | Brazil | 51 | 0 | 0 | 51 | NA | NA | NA | NA | NA | NA | NA | NA | 21.29 | NA | NA | | 6.4 | NA | NA | 28 | NA | NA | NA | 28 | NA | NA | NA |
| Thohar Arifin et al.,2020 | retrospective cohort | Indonesia | 16 | 9 | 7 | 0 | NA | NA | NA | NA | 1NA6 | NA | 12.56 | 13.71 | NA | 5.38 | 7.04 | | NA | 5 | 1 | NA | NA | NA | NA | NA | NA | NA | NA |
| Kanai et al.,2021 | retrospective cohort | Japan | 17 | 14 | 3 | NA | NA | NA | NA | NA | NA | NA | NA | NA | NA | 2.92 | 3 | | NA | 6 | 1 | NA | Engel  class | NA | NA | NA | NA | NA | NA |
| Duc Lien et al.,2020 | retrospective cohort | Vietnam | 16 | 11 | 5 | NA | NA | NA | NA | NA | NA | NA | 4.18 | 7.8 | NA | 2.9 | 3.47 | | NA | 0 | 1 | NA | NA | NA | NA | NA | NA | NA | NA |
| Ueda et al.,2019 | retrospective cohort | Japan | 21 | NA | NA | NA | NA | NA | NA | NA | NA | NA | 8.6 | | NA | 1 | 1 | | NA | 4 | | NA | NA | NA | NA | NA | NA | NA | NA |
| Baba et al.,2018 | retrospective cohort | Japan | 56 | 54 | 2 | NA | NA | NA | NA | NA | NA | NA | 1.88 | | NA | 3.05 | | | NA | 18 | | NA | NA | NA | NA | NA | NA | NA | NA |
| Paglioli et al.,2016 | prospective cohort | Brazil | 36 | 0 | 0 | 36 | NA | NA | 36 | NA | NA | 24NA12 | NA | NA | 21.5±11.3 | NA | NA | | 69  (106.8) | NA | NA | 24 | NA | NA | NA | 24 | NA | NA | 0 |
| Iwasaki et al.,2016 | retrospective cohort | Japan | 26 | 26 | 0 | 0 | 22 | NA | NA | 12/14 | NA | NA | 8.8 | NA | NA | 2.83 | NA | | NA | 5 | NA | NA | Engel  class | 15 | NA | NA | NA | NA | NA |
| Otsuki et al.,2015 | prospective cohort | Japan | 30 | 28 | 2 | NA | NA | NA | NA | NA | NA | NA | NA | NA | NA | NA | NA | | NA | 9 | | NA | Kaplan–Meier analysis | NA | NA | NA | NA | NA | NA |
| Liang et al.,2015 | retrospective cohort | China | 14 | 8 | 6 | NA | 9 | | NA | NA | NA | NA | 12.64±8.54 | | NA | NA | NA | | NA | 4 | | NA | NA | 5 | | NA | 1 | | NA |
| Yang et al.,2014 | retrospective cohort | China | 29 | 0 | 29 | 0 | NA | 29 | NA | NA | 19/10 | NA | NA | 9.96  (4.5) | NA | NA | 5.2（NA) | | NA | NA | NA | NA | NA | NA | 15 | NA | NA | 23 | NA |
| Stigsdotter-Broman et al.,2014 | prospective cohort | Sweden | 31 | 11 | 19 | 1 | 18 | | | 20/11 | | | 13.3 | | | NA | NA | | NA | 2 | | | NA | 10 | | | NA | NA | NA |
| Passamonti et al.,2014 | retrospective cohort | Italy | 26 | 9 | 17 | 0 | NA |  |  | NA | NA | NA | 25(8) |  |  | 14  (8) |  | |  | 2 | | NA | McHugh class | 11 | | NA | NA | NA | NA |
| Liang et al.,2014 | prospective cohort | China | 23 | NA | 23 | NA | NA | NA | NA | NA | NA | NA | NA | 9.48  (2.21) | NA | NA | NA | | NA | NA | 4;3;2 | NA | Engel  class | NA | NA | NA | NA | 4 | NA |
| Cukiert et al.,2013 | retrospective cohort | Brazil | 24 | NA | 24 | NA | NA | NA | NA | NA | 11/9 | NA | NA | 8.63 | NA | NA | NA | | NA | NA | 2 | NA | NA | NA | NA | NA | NA | 23 | NA |
| Asadi-Pooya et al.,2013 | retrospective cohort | Iran | 18 | NA | 18 | NA | NA | 11 | NA | NA | 14/4 | NA | NA | 9.9 | NA | NA | 1.88 | | NA | NA | 3 | NA | NA | NA | 4 | NA | NA | 1 | NA |
| Lin et al.,2012 | retrospective cohort | China | 48 | NA | 48 | NA | NA | NA | NA | NA | 33/15 | NA | NA | 7.6 | NA | NA | 5.8 | | NA | NA | 0 | NA | NA | NA | NA | NA | NA | NA | NA |
| Liang et al.,2010 | prospective cohort | China | 60 | NA | 60 | NA | NA | NA | NA | NA | 39/21 | NA | NA | NA | NA | NA | NA | | NA | NA | 5 | NA | NA | NA | NA | NA | NA | NA | NA |
| Tanriverdi et al.,2009 | retrospective cohort | Canada | 95 | 12 | 83 | NA | 79 | | NA | 49/46 | | NA | 24.03（10.5） | | NA | 17.2（5.09） | | | NA | 0 | | NA | NA | 44.30%（35/79） | |  | 0 | 0 | NA |
| Sunaga et al.,2009 | retrospective cohort | Japan | 78 | 63 | 15 | NA | 60 | 13 | NA | 50/28 | | NA | 14.4 | | NA | 7（2.9） | | |  | NA | | NA | NA | 54 | 7 | NA | 2 | | NA |
| Ping et al.,2009 | retrospective cohort | China | 31 | 0 | 31 | NA | 9 | NA | NA | NA | 17/14 | NA | NA | 17.5 | NA | NA | 3 | | NA | NA | 12 | NA | Engel  class | NA | NA | NA | NA | NA | NA |
| Cukiert et al.,2009 | retrospective cohort | Brazil | 11 | 0 | 11 | NA | NA | NA | NA | NA | 8/3 | NA | NA | 31.55  (9.43) | NA | NA | 4.08 | | NA | NA | 0 | NA | NA | NA | 2 | NA | NA | 0 | NA |
| You et al.,2008 | retrospective cohort | Korea | 14 | 14 | 0 | NA | NA | NA | NA | 10/4 | NA | NA | NA | NA | NA | 3.07  (2.93) | NA | | NA | 4 | NA | NA | NA | NA | NA | NA | 0 | NA | NA |
| Shim et al.,2008 | retrospective cohort | Korea | 34 | 34 | 0 | NA | 34 | 0 | NA | 22/2 | NA | NA | 8.7 | NA | NA | 2.58 | NA | | NA | 12 | NA | NA | Engel  class | 25 | NA | NA | 2 | NA | NA |
| Rathore et al.,2007 | retrospective cohort | India | 17 | 11 | 6 | NA | 11 | 6 | NA | 10/1 | 6/0 | NA | 8.02(3.1) | 13  (2.52) | NA | 4.7 | | | NA | 2 | 0 | NA | Engel  class | 6 | 1 | NA | 0 | 0 | NA |
| Turanli et al.,2006 | retrospective cohort | Turkey | 16 | 0 | 16 | 0 | 0 | 13 | NA | NA | NA | NA | NA | 7.4 | NA | NA | 7.3 | | NA | NA | 1 | NA | NA | NA | 6 | NA | NA | 0 | NA |
| Kwan et al.,2006 | retrospective cohort | China | 74 | NA | 74 | NA | NA | NA | NA | NA | 53/21 | NA | NA | 9.1 | NA | NA | NA | | NA | NA | 0 | NA | NA | NA | NA | NA | NA | NA | NA |
| Cukiert et al.,2006 | retrospective cohort | Brazil | 76 | NA | 76 | NA | NA | NA | NA | NA | 42/34 | NA | NA | 11.2 | NA | NA | 4.7 | | NA | NA | 7 | NA | NA | NA | NA | NA | NA | 72 | NA |
| Shimizu et al.,2005 | retrospective cohort | Japan | 76 | 76 | | NA | 35 | 6 | NA | NA | NA | NA | NA | NA | NA | NA | NA | | NA | NA | NA | NA | Engel class | 32 | 4 | NA | NA | NA | NA |
| Kim et al.,2004 | retrospective cohort | Korea | 21 | 4 | 17 | NA | 21 | | NA | 12/9 | | NA | 19.4 | | NA | 4.75 | | | NA | 0 | 4 | NA | Modified Wyler's Classification | 0 | 12 | NA | 0 | 4 | NA |
| Kawai et al.,2004 | retrospective cohort | Japan | 10 | 10 | NA | NA | 10 | NA | NA | 4/6 | NA | NA | 12.91 | NA | NA | 3.13 | NA | | NA | 1 | NA | NA | NA | 8 | NA | NA | NA | NA | NA |
| Taketoshi Maehara and Hiroyuki Shimizu,2001 | retrospective cohort | Japan | 52 | 35 | 17 | NA | 35 | 17 | NA | 34/18 | |  | 18^#^ | | NA | 3.33 | | | NA | 0 | 0 | NA | NA | NA | NA | NA | NA | NA | NA |
| Kwan et al.,2001 | retrospective cohort | China | 61 | NA | 61 | NA | NA | NA | NA | NA | 47/14 | NA | NA | NA | NA | NA | NA | | NA | NA | 0 | NA | NA | NA | NA | NA | NA | NA | NA |
| Fandiño-Franky et al.,2001 | retrospective cohort | | 95 | NA | 95 | NA | NA | NA | NA | NA | NA | NA | NA | NA | NA | NA | NA | | NA | NA | 9 | NA | NA | NA | 11 | NA | NA | NA | NA |
| Carmant et al.,1998 | retrospective cohort | Canada | 28 | 28 | | NA | NA | NA | NA | NA | NA | NA | 13.8 | | NA | 2.8 | | | NA | 0 | | NA | NA | 1 | | NA | 3 | | NA |
| Sakas et al.,1997 | retrospective cohort | Ireland | 20 | NA | 20 | NA | NA | 13 | NA | NA | 14/6 | NA | NA | 25.7 | NA | NA | 6.7 | | NA | NA | 6 | NA | NA | NA | 6 | NA | NA | 3 | NA |
| Rossi et al.,1996 | retrospective cohort | Italy | 20 | 2 | 18 | NA | NA | NA | NA | 14/6 | | NA | 23 | | NA | 3.5 | | | NA | 2 | | NA | NA | NA | NA | NA | 10 | | NA |
| Andersen et al.,1996 | retrospective cohort | Denmark | 20 | 9 | 11 | NA | NA | NA | NA | 13/7 | | NA | 20.8 | | NA | NA | 3.25 | | NA | NA | 3 | NA | NA | NA | NA | NA | 3 | 9 | NA |
| Mamelak et al.,1993 | retrospective cohort | America | 15 | 5 | 10 | NA | NA | NA | NA | 2/3 | 6/4 | NA | 21 | 23.7 | NA | a minimum follow-up of one year | | | NA | 1 | 6 | NA | NA | NA | NA | NA | 0 | 0 | NA |
| Cendes et al.,1992 | retrospective cohort | Brazil | 34 | 13 | 12 | 9 | MA | MA | MA | MA | MA | MA | 7.98 | 11.58 | 7.44 | 3.5 | | | | 0 | 0 | 0 | NA | NA | NA | NA | 5 | 0 | 0 |
| Oguni et al.,1991 | retrospective cohort | Canada | 43 | NA | 43 | NA | NA | 31 | NA | NA | 20/23 | NA | NA | 23.5 | NA | NA | 3.25 | | NA | NA | 5 | NA | NA | NA | 14 | NA | NA | 0 | NA |
| Nordgren et al.,1991 | retrospective cohort | Germany | 15 | 13 | 2 | NA | NA | NA | NA | 8/5 | 2/0 | NA | 12.38 | 14.5 | NA | NA | NA | | NA | 1 | 0 | NA | NA | NA | NA | NA | 0 | 0 | NA |
| Spencer et al.,1991 | retrospective cohort | America | 25 | 6 | 19 | NA | NA | NA | NA | NA | NA | NA | NA | NA | NA | 3.89 | 5.17 | | NA | 0 | 0 | NA | NA | NA | NA | NA | NA | NA | NA |
| Cohen et al.,1991 | retrospective cohort | America | 10 | NA | 10 | NA | NA | NA | NA | NA | 6/4 | NA | NA | 7.1 | NA | NA | NA | | NA | NA | 1 | NA | NA | NA | NA | NA | NA | NA | NA |
| Makari et al.,1989 | retrospective cohort | America | 20 | NA | 20 | NA | NA | NA | NA | NA | 13/7 | NA | NA | 15.2 | NA | NA | 2.59 | | NA | NA | 2 | NA | NA | NA | NA | NA | NA | NA | NA |
| Gates Júnior et al.,1987 | retrospective cohort | America | 24 | NA | 24 | NA | NA | NA | NA | NA | 16/8 | NA | NA | NA | NA | NA | 3.59 | | NA | NA | 3 | NA | NA | NA | NA | NA | NA | NA | NA |

NA, not reported; Age at surgery is presented as means, except for marked with ^#^.
