# Supplementary material for: Long‐term follow‐up seizure outcomes after corpus callosotomy: A systematic review with meta‐analysis
Source: Brain Behav. 2023 Mar 16;13(4):e2964. doi: 10.1002/brb3.2964 (PMC10097058; doi:10.1002/brb3.2964)
Supplement: Supplementary file 3 — Supplementary Materials 3. The quality of the case series studies was assessed by part of the METHODOLOGICAL INDEX FOR NON‐RANDOMIZED STUDIES (MINORS) Scale [file BRB3-13-e2964-s004.docx]

**Supplementary Materials 3. The quality of the case series studies was assessed by part of the METHODOLOGICAL INDEX FOR NON-RANDOMIZED STUDIES (MINORS) Scale**

| Study | A clearly stated aim | Inclusion of consecutive patients | Prospective collection of data | Endpoints appropriate to the aim of the study | Unbiased evaluation of endpoints | Follow-up period appropriate to the major endpoint | Loss to follow up not exceeding 5% | Prospective calculation of the study size | Total |
| --- | --- | --- | --- | --- | --- | --- | --- | --- | --- |
| Ferrand-Sorbets et al.,2022 | 2 | 1 | 2 | 2 | 2 | 2 | 2 | 0 | 13 |
| Na et al.,2022 | 2 | 2 | 2 | 2 | 0 | 2 | 2 | 0 | 12 |
| Sadashiva et al.,2022 | 2 | 1 | 2 | 2 | 0 | 2 | 2 | 0 | 11 |
| Ukishiro et al.,2022 | 2 | 1 | 2 | 2 | 2 | 2 | 2 | 0 | 13 |
| Kagawa et al.,2021 | 2 | 1 | 2 | 2 | 0 | 2 | 2 | 0 | 11 |
| Honda et al.,2021 | 2 | 1 | 2 | 2 | 0 | 2 | 2 | 0 | 11 |
| Frigeri et al.,2021 | 2 | 1 | 2 | 2 | 2 | 2 | 2 | 0 | 13 |
| Thohar Arifin et al.,2020 | 2 | 1 | 2 | 2 | 0 | 2 | 2 | 0 | 11 |
| Kanai et al.,2021 | 2 | 1 | 2 | 2 | 0 | 2 | 2 | 0 | 11 |
| Duc Lien et al.,2020 | 2 | 1 | 2 | 2 | 2 | 2 | 2 | 0 | 13 |
| Ueda et al.,2019 | 2 | 1 | 2 | 2 | 2 | 2 | 2 | 0 | 13 |
| Baba et al.,2018 | 2 | 2 | 2 | 2 | 0 | 2 | 2 | 0 | 12 |
| Paglioli et al.,2016 | 2 | 0 | 2 | 2 | 0 | 2 | 2 | 0 | 10 |
| Iwasaki et al.,2016 | 2 | 2 | 2 | 2 | 0 | 2 | 2 | 0 | 12 |
| Otsuki et al.,2015 | 2 | 1 | 2 | 2 | 0 | 2 | 2 | 0 | 11 |
| Liang et al.,2015 | 2 | 1 | 2 | 2 | 0 | 2 | 2 | 0 | 11 |
| Yang et al.,2014 | 2 | 2 | 2 | 2 | 0 | 2 | 2 | 0 | 12 |
| Stigsdotter-Broman et al.,2014 | 2 | 0 | 1 | 2 | 0 | 2 | 2 | 0 | 9 |
| Passamonti et al.,2014 | 2 | 1 | 1 | 2 | 0 | 2 | 2 | 0 | 10 |
| Liang et al.,2014 | 2 | 1 | 2 | 2 | 0 | 2 | 2 | 0 | 11 |
| Cukiert et al.,2013 | 2 | 1 | 2 | 2 | 0 | 2 | 2 | 0 | 11 |
| Asadi-Pooya et al.,2013 | 2 | 1 | 2 | 2 | 0 | 2 | 2 | 0 | 11 |
| Lin et al.,2012 | 2 | 1 | 2 | 2 | 2 | 2 | 2 | 0 | 13 |
| Liang et al.,2010 | 2 | 1 | 2 | 2 | 2 | 2 | 2 | 0 | 13 |
| Tanriverdi et al.,2009 | 2 | 2 | 2 | 2 | 0 | 2 | 2 | 0 | 12 |
| Sunaga et al.,2009 | 2 | 1 | 2 | 2 | 0 | 2 | 2 | 0 | 11 |
| Ping et al.,2009 | 2 | 1 | 2 | 2 | 0 | 2 | 2 | 0 | 11 |
| Cukiert et al.,2009 | 2 | 1 | 2 | 2 | 2 | 2 | 2 | 0 | 13 |
| You et al.,2008 | 2 | 1 | 2 | 2 | 2 | 2 | 2 | 0 | 13 |
| Shim et al.,2008 | 2 | 2 | 2 | 2 | 0 | 2 | 2 | 0 | 12 |
| Rathore et al.,2007 | 2 | 0 | 0 | 2 | 0 | 2 | 2 | 0 | 8 |
| Turanli et al.,2006 | 2 | 2 | 2 | 2 | 0 | 2 | 2 | 0 | 12 |
| Kwan et al.,2006 | 2 | 0 | 2 | 2 | 0 | 2 | 2 | 0 | 10 |
| Cukiert et al.,2006 | 2 | 2 | 2 | 2 | 0 | 2 | 2 | 0 | 12 |
| Shimizu et al.,2005 | 2 | 1 | 1 | 2 | 0 | 2 | 2 | 0 | 10 |
| Kim et al.,2004 | 2 | 1 | 2 | 2 | 0 | 2 | 2 | 0 | 11 |
| Kawai et al.,2004 | 2 | 2 | 2 | 2 | 0 | 2 | 2 | 0 | 12 |
| Taketoshi Maehara and Hiroyuki Shimizu,2001 | 2 | 1 | 2 | 2 | 0 | 2 | 2 | 0 | 11 |
| Kwan et al.,2001 | 2 | 2 | 2 | 2 | 0 | 2 | 2 | 0 | 12 |
| Fandiño-Franky et al.,2001 | 2 | 0 | 2 | 2 | 0 | 2 | 2 | 0 | 10 |
| Carmant et al.,1998 | 2 | 1 | 1 | 2 | 0 | 2 | 2 | 0 | 10 |
| Sakas et al.,1997 | 2 | 0 | 1 | 2 | 0 | 2 | 2 | 0 | 9 |
| Rossi et al.,1996 | 2 | 1 | 1 | 2 | 0 | 2 | 2 | 0 | 10 |
| Andersen et al.,1996 | 2 | 0 | 0 | 2 | 0 | 2 | 2 | 0 | 8 |
| Mamelak et al.,1993 | 2 | 1 | 1 | 2 | 0 | 2 | 2 | 0 | 10 |
| Cendes et al.,1992 | 2 | 0 | 2 | 2 | 0 | 2 | 2 | 0 | 10 |
| Oguni et al.,1991 | 2 | 0 | 2 | 2 | 0 | 2 | 2 | 0 | 10 |
| Nordgren et al.,1991 | 2 | 2 | 2 | 2 | 0 | 2 | 2 | 0 | 12 |
| Spencer et al.,1991 | 2 | 1 | 1 | 2 | 0 | 2 | 2 | 0 | 10 |
| Cohen et al.,1991 | 2 | 0 | 1 | 2 | 0 | 2 | 2 | 0 | 9 |
| Makari et al.,1989 | 2 | 1 | 1 | 2 | 0 | 2 | 2 | 0 | 10 |
| Gates Júnior et al.,1987 | 2 | 0 | 0 | 2 | 0 | 2 | 2 | 0 | 8 |
