# Supplementary figures and images for: Long‐term follow‐up seizure outcomes after corpus callosotomy: A systematic review with meta‐analysis
Source: Brain Behav. 2023 Mar 16;13(4):e2964. doi: 10.1002/brb3.2964 (PMC10097058; doi:10.1002/brb3.2964)

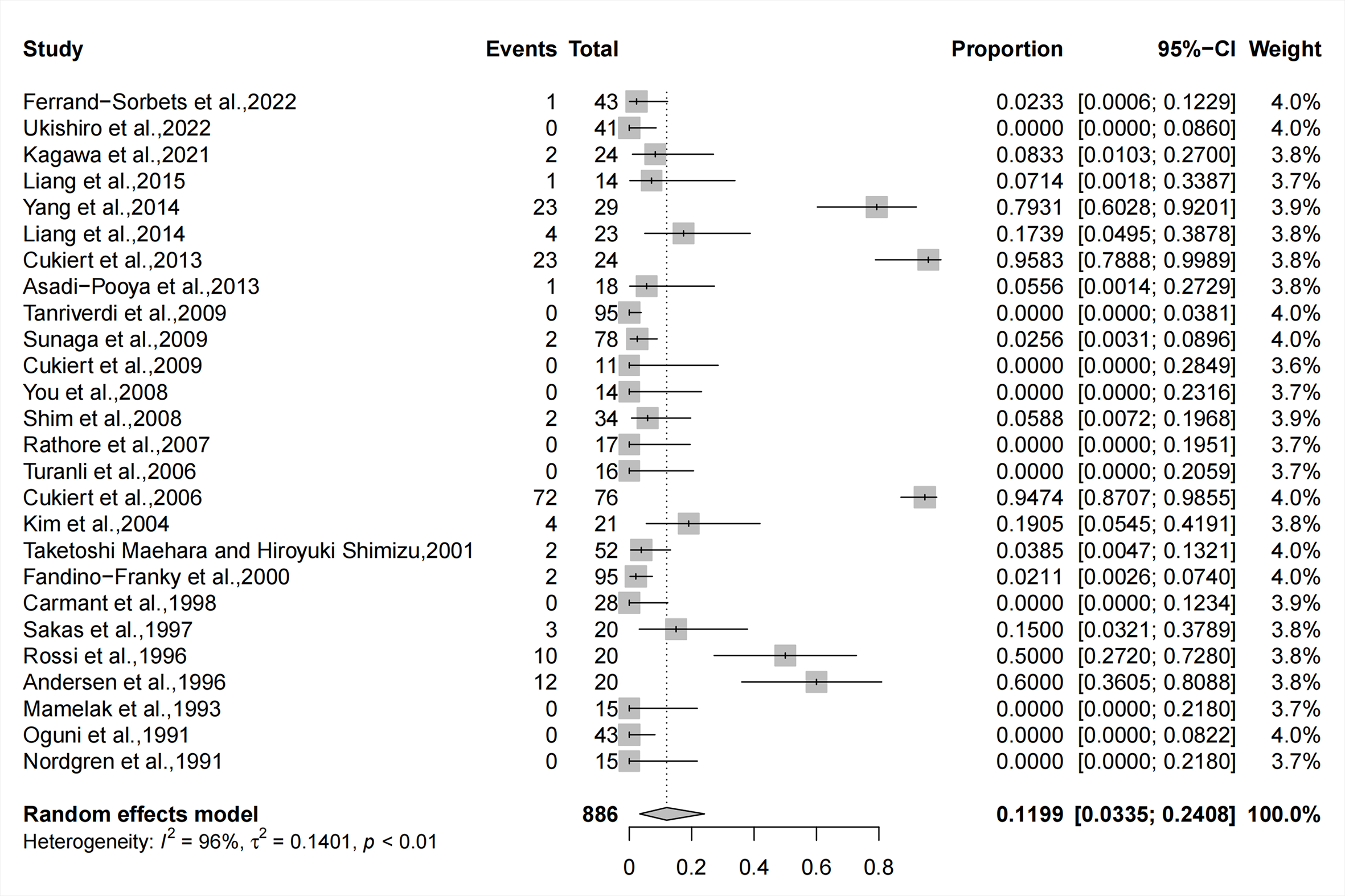

Supplement: Supplementary file 4 — Supplementary Materials 4 [file BRB3-13-e2964-s002.tif]

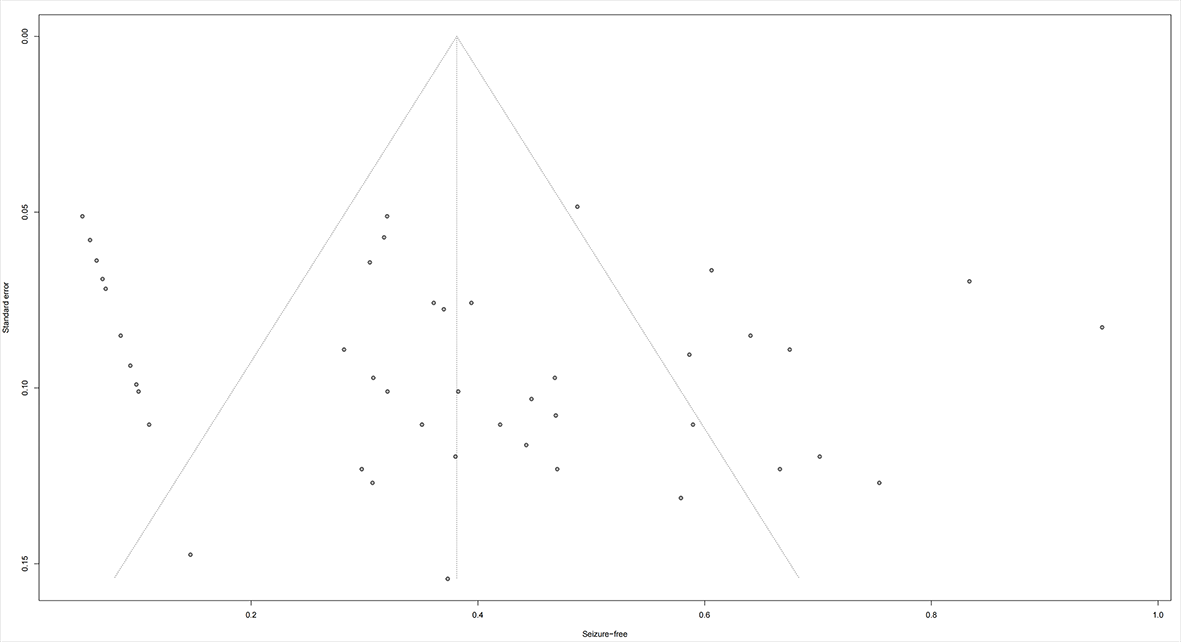

Supplement: Supplementary file 5 — Supplementary Materials 5 [file BRB3-13-e2964-s005.tif]
