# Supplementary material for: Long‐term follow‐up seizure outcomes after corpus callosotomy: A systematic review with meta‐analysis
Source: Brain Behav. 2023 Mar 16;13(4):e2964. doi: 10.1002/brb3.2964 (PMC10097058; doi:10.1002/brb3.2964)
Supplement: Supplementary file 6 — Supplementary Materials 6. GRADE Assessment [file BRB3-13-e2964-s001.docx]

**Supplementary Materials 6. GRADE Assessment**

| **Patients (n)** | **Effect (95% CI)** | **Certainty assessment** | | | | | | | **Certainty** | **Importance** |
| --- | --- | --- | --- | --- | --- | --- | --- | --- | --- | --- |
|  |  | **Studies (n)** | **Design** | **Risk of bias** | **Inconsistency** | **Indirectness** | **Imprecision** | **Publication bias** |  |  |
| **The rate of complete seizure freedom** | | | | | | | | | | |
| 1644 | 12.38% (95% CI 8.17–17.21%) | 49 | retrospective cohort | not serious | serious | not serious | serious | none | Moderate | CRITICAL |
| **The rate of complete SF from drop attacks** | | | | | | | | | | |
| 587 | 61.86% (95% CI 51.87–71.41%) | 20 | retrospective cohort | not serious | serious | not serious | serious | none | Moderate | CRITICAL |
| **The rate of acute disconnection syndrome** | | | | | | | | | | |
| 886 | 11.99% (95% CI 3.35–24.08%) | 26 | retrospective cohort | not serious | serious | not serious | serious | none | Low | CRITICAL |
